# Prenatal diagnosis by trio exome sequencing in fetuses with ultrasound anomalies: A powerful diagnostic tool

**DOI:** 10.3389/fgene.2023.1099995

**Published:** 2023-03-23

**Authors:** Frédéric Tran Mau-Them, Julian Delanne, Anne-Sophie Denommé-Pichon, Hana Safraou, Ange-Line Bruel, Antonio Vitobello, Aurore Garde, Sophie Nambot, Nicolas Bourgon, Caroline Racine, Arthur Sorlin, Sébastien Moutton, Nathalie Marle, Thierry Rousseau, Paul Sagot, Emmanuel Simon, Catherine Vincent-Delorme, Odile Boute, Cindy Colson, Florence Petit, Marine Legendre, Sophie Naudion, Caroline Rooryck, Clément Prouteau, Estelle Colin, Agnès Guichet, Alban Ziegler, Dominique Bonneau, Godelieve Morel, Mélanie Fradin, Alinoé Lavillaureix, Chloé Quelin, Laurent Pasquier, Sylvie Odent, Gabriella Vera, Alice Goldenberg, Anne-Marie Guerrot, Anne-Claire Brehin, Audrey Putoux, Jocelyne Attia, Carine Abel, Patricia Blanchet, Constance F. Wells, Caroline Deiller, Mathilde Nizon, Sandra Mercier, Marie Vincent, Bertrand Isidor, Jeanne Amiel, Rodolphe Dard, Manon Godin, Nicolas Gruchy, Médéric Jeanne, Elise Schaeffer, Pierre-Yves Maillard, Frédérique Payet, Marie-Line Jacquemont, Christine Francannet, Sabine Sigaudy, Marine Bergot, Emilie Tisserant, Marie-Laure Ascencio, Christine Binquet, Yannis Duffourd, Christophe Philippe, Laurence Faivre, Christel Thauvin-Robinet

**Affiliations:** ^1^ Unité Fonctionnelle Innovation en Diagnostic Génomique des Maladies Rares, CHU Dijon Bourgogne, Dijon, France; ^2^ INSERM UMR1231 GAD, F-21000, Dijon, France; ^3^ Centre de Référence Maladies Rares “Anomalies Du Développement et Syndromes Malformatifs”, Centre de Génétique, FHU TRANSLAD et Institut GIMI, CHU Dijon Bourgogne, Dijon, France; ^4^ Laboratoire Génétique Chromosomique et Moléculaire, CHU Dijon Bourgogne, Dijon, France; ^5^ Service de Gynécologie Obstétrique, Médecine Fœtale et Stérilité Conjugale, Centre Hospitalier Universitaire Dijon Bourgogne, Dijon, France; ^6^ CHU Lille, Clinique de Génétique Guy Fontaine, Centre de Référence Maladies Rares “Anomalies Du Développement et Syndromes Malformatifs” Nord-Ouest, FLille, France; ^7^ CHU de Bordeaux, Service de Génétique Médicale, Bordeaux, France; ^8^ Biochemistry and Genetics Department, University Hospital of Angers, Angers, France; ^9^ Service de Génétique Clinique, Centre de Référence Maladies Rares CLAD-Ouest, CHU Hôpital Sud, Rennes, France; ^10^ Service de Génétique—Unité de Génétique Clinique, Rouen, France; ^11^ Service de Génétique—GH Est-Hôpital Femme Mère Enfant, Lyon, France; ^12^ Service de Gynécologie-obstétrique, HCL, Lyon, France; ^13^ Service de Génétique et Centre de Diagnostic Anténatal, CHU de Lyon HCL—GH Nord-Hôpital de La Croix Rousse, Lyon, France; ^14^ Equipe Maladies Génétiques de L’Enfant et de L’Adulte, Département Génétique Médicale, Maladies Rares et Médecine Personnalisée, CHU de Montpellier, University Montpellier, Montpellier, France; ^15^ CHU Nantes, Service de Génétique Médicale, Nantes, France; ^16^ Institut Du Thorax, INSERM, CNRS, UNIV Nantes, Nantes, France; ^17^ Equipe “Embryologie et Génétiques des Malformations Congénitales", Institut Imagine—INSERM U1163, Institut des Maladies Génétiques, Paris, France; ^18^ Service de Génétique Médicale et Clinique, Hôpital Necker-Enfants Malades, Paris, France; ^19^ Unité Fonctionnelle de Génétique Médicale, Cytogénétique, Génétique Médicale et Biologie de La Reproduction, Centre Hospitalier Intercommunal Poissy-Saint-Germain-en-Laye, Poissy, France; ^20^ Service de Génétique, CHU Caen Clemenceau, EA 7450 Biotargen, University Caen, Caen, France; ^21^ Service de Génétique, CHU de Tours, Tours, France; ^22^ UMR 1253, IBrain, Université de Tours, Inserm, Tours, France; ^23^ Service de Génétique Médicale, CHU de Strasbourg—Hôpital de Hautepierre, Strasbourg, France; ^24^ Service de Génétique Médicale, Pôle Femme, Mère, Enfants CHU de La Réunion—GH Sud Réunion—Saint-Pierre, Saint-Pierre, France; ^25^ Service de Génétique Médicale, Pôle Femme et Enfant, CHU de Clermont-Ferrand—Hôpital D'Estaing, Clermont-Ferrand, France; ^26^ Unité de Génétique Clinique Prénatale, Département de Génétique Médicale, CHU de Marseille—Hôpital de La Timone, Marseille, France; ^27^ Centre D'Investigation Clinique CIC-EC Inserm CIC1432, UFR des Sciences de Santé, Université de Bourgogne-Franche-Comté, Dijon, France

**Keywords:** exome sequencing (ES), chromosomal microarray, prenatal, fetal, diagnostic yield

## Abstract

**Introduction:** Prenatal ultrasound (US) anomalies are detected in around 5%–10% of pregnancies. In prenatal diagnosis, exome sequencing (ES) diagnostic yield ranges from 6% to 80% depending on the inclusion criteria. We describe the first French national multicenter pilot study aiming to implement ES in prenatal diagnosis following the detection of anomalies on US.

**Patients and methods:** We prospectively performed prenatal trio-ES in 150 fetuses with at least two US anomalies or one US anomaly known to be frequently linked to a genetic disorder. Trio-ES was only performed if the results could influence pregnancy management. Chromosomal microarray (CMA) was performed before or in parallel.

**Results:** A causal diagnosis was identified in 52/150 fetuses (34%) with a median time to diagnosis of 28 days, which rose to 56/150 fetuses (37%) after additional investigation. Sporadic occurrences were identified in 34/56 (60%) fetuses and unfavorable vital and/or neurodevelopmental prognosis was made in 13/56 (24%) fetuses. The overall diagnostic yield was 41% (37/89) with first-line trio-ES *versus* 31% (19/61) after normal CMA. Trio-ES and CMA were systematically concordant for identification of pathogenic CNV.

**Conclusion:** Trio-ES provided a substantial prenatal diagnostic yield, similar to postnatal diagnosis with a median turnaround of approximately 1 month, supporting its routine implementation during the detection of prenatal US anomalies.

## 1 Introduction

Isolated or multiple congenital anomalies (MCA) affect around 2% of pregnancies, possibly secondary to maternal etiologies (placental, infectious, toxic) but mainly due to genetic disorders (Wojcik et al., 2019). These disorders are a genuine medical challenge, particularly because of their perinatal mortality that is around 20% (Best et al., 2018; Normand et al., 2018). The rapid identification of a causal diagnosis is therefore essential for adapting prenatal/perinatal management and providing genetic counseling for the current pregnancy and any subsequent pregnancies. According to the recommendations of the American College of Obstetricians and Gynecologists, current prenatal genetic investigations are based on the standard karyotype and chromosomal microarray analysis (CMA) in fetuses with one or more US anomalies (Committee on Genetics and the Society for Maternal-Fetal Medicine; Committee Opinion no. 2016). When a causal diagnosis is suspected on US anomalies, targeted gene sequencing or fluorescence *in situ* hybridization can also be performed. The CMA identifies causal diagnosis in around 6% of fetuses with US anomalies and normal karyotype (Levy and Wapner, 2018). Despite CMA and targeted analyses, about 70% of fetuses with MCA remain without molecular diagnosis (Best et al., 2018; Normand et al., 2018).

In the last decade, exome/genome sequencing (ES/GS) became the first-tier genetic test for the causal diagnosis of individuals with congenital anomalies. Postnatal clinical ES yield ranges from 30% to 50% depending on the clinical cohort and the strategy used (solo/trio) (Clark et al., 2018). Few countries have implemented or performed ES in prenatal settings, with a highly variable yield, ranging from 6% to 92% (Best et al., 2018; Ferretti et al., 2019; Guadagnolo et al., 2021). This variation reflects the heterogeneity of the fetal cohorts (which includes MCA or isolated malformations), the ES strategy (solo or trio), and on tests performed before ES (CMA, panel sequencing) (Diderich et al., 2021). Many challenges have to be overcome before ES can be used routinely in prenatal diagnosis, such as variant interpretation on partial phenotypes mostly based on imagery (US, X-ray, and/or magnetic resonance imaging), the poor prenatal description of Mendelian disorders (Aggarwal et al., 2020), and timing constraints inherent to the ongoing pregnancy.

When MCA is detected using prenatal US, a rapid etiological diagnosis can clarify the prognosis and help with decision-making, i.e., medical termination of pregnancy (ToP) or conservative procedures, as well as perinatal management. Here, we report the first French national pilot study of trio-ES in prenatal diagnosis. We evaluate the feasibility of delivering a result in less than 4 weeks for being compatible with pregnancy management, identify the technical or organizational obstacles, and evaluate the diagnostic yield of first-line trio-ES or after CMA and the effect on the continuation and monitoring of pregnancy.

## 2 Patients and methods

### 2.1 Patients

Pregnancies [10–32 weeks of gestation (WG)] were prospectively included following the detection of US anomalies, specifically i) two major anomalies, ii) one major and one minor anomaly, or iii) one anomaly (major or minor) with a strong suspicion of genetic cause (such as *corpus callosum* anomaly). Isolated nuchal translucency and hygroma were excluded. The definition of major and minor anomalies was based on a previous publication (DeSilva et al., 2016). Abnormalities were considered major if they had an impact on life expectancy, health status, and physical or social functioning (not applicable in prenatal settings) (DeSilva et al., 2016). On the contrary, abnormalities were considered minor when they had little or no impact on health and functioning. Because the identification of a causal diagnosis was intended to help in parental decision-making about the pregnancy, couples with an immediate referral for ToP were not included. Appropriate written consent was obtained from all participants in accordance with the ethics committee that approved the ANDDI-PRENATOME study (NCT03964441). The clinical features were collected in an electronic case report form devoted to the study and by using Human Phenotype Ontology (HPO). Routine CMA was performed before or concomitantly to trio-ES. When ES was performed concomitantly to CMA, we referred to this strategy as the first-line (FL), and when ES was performed after CMA, we referred to it as the second-line (SL).

### 2.2 Exome sequencing and variant interpretation

Exome sequencing (ES) was performed using a trio-based strategy (fetus and both parents) from DNA extracted from amniotic fluid (15 ml) or fetal blood samples (6 ml) and parental blood samples (10 ml). The FastQ file generation was outsourced to a single sequencing platform (from DNA to raw data) and performed on a NovaSeq 6000 device (Illumina) with the enriched version of the TWIST-HCE (Human Core Exome) Kit (Twist Bioscience), according to the supplier's protocol. Vcf files were generated with the local bioinformatics solution (Tran Mau-Them et al., 2021). CNV detection was done with an Exome Hidden Markov Model (XHMM) (Fromer et al., 2012).

Each selected variant was ranked into one of the five categories from the ACMG recommendations (Richards et al., 2015). The variants that were considered pathogenic and likely pathogenic were returned to the referring clinicians, as were some variants of uncertain significance (VUS), when the multidisciplinary team considered that their implication in the phenotype was very likely and/or when additional investigations and/or family segregation could be performed to confirm or exclude the pathogenicity of the variants.

Variant confirmation and parental segregation were confirmed by Sanger sequencing (primers and conditions available on request). CNV was confirmed by qPCR (primers and conditions available on request) unless identified by CMA.

### 2.3 Analytical stages and pregnancy issues

To evaluate the time required to return the results (with confirmation for positives and without confirmation for negatives), we considered the day of arrival of the three samples (fetus and both parents) at the laboratory as day 0, since we observed outliers caused by shipment duration or delays in the reception of parental samples that were independent from our laboratory. At every step of the process, durations were measured from D0 to the end point (day of emailed molecular report), namely, from *reception* to *outsourcing*, from *outsourcing* to *raw data reception*, from *raw data reception* to *vcf generation*, from *vcf generation* to *multidisciplinary meeting* (MDM), from MDM to *Sanger sequencing* (if variant retained), and from *Sanger sequencing* to *molecular report* (end point). The fetal prognosis issued of molecular diagnosis, as well as of pregnancy outcome, was systematically collected.

## 3 Results

### 3.1 Patients

**FIGURE 1:**
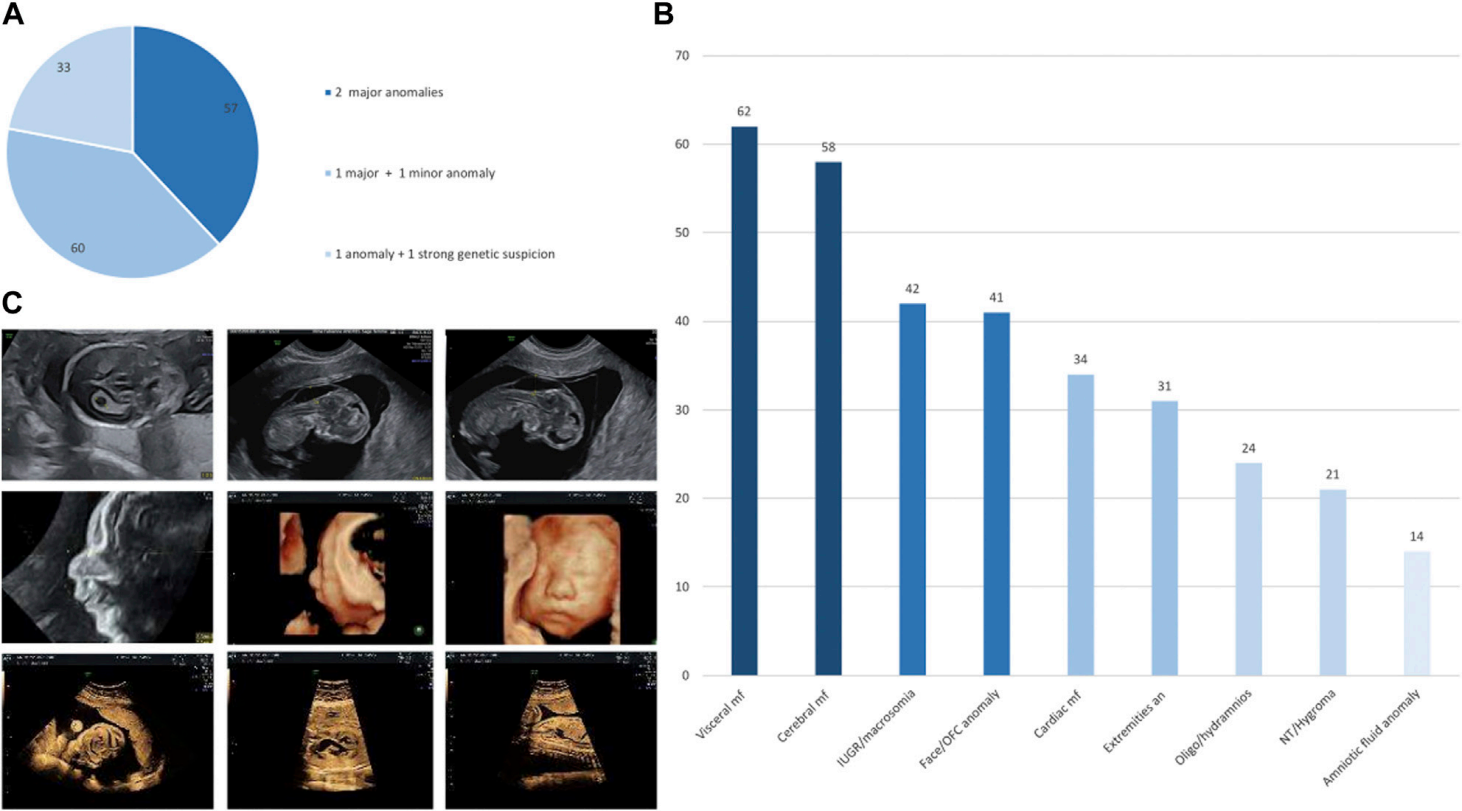
**(A)** Percentage of fetuses included in the defined subgroups, namely, two major anomalies, one major and one minor anomaly, and one anomaly with a strong suspicion of genetic disorder. **(B)** Histograms of the distribution of anomalies in the entire cohort, ranging from the most frequent sign on the left to the most uncommon sign on the right. IUGR, intra uterine growth restriction; NT, nuchal translucency; OFC, occipitofrontal circumference. **(C)** Ultrasonography images of the fetus referred for persistent increased nuchal translucency with pathogenic truncating homozygous *ASCC1* variant associated with a truncating homozygous variant of unknown significance in *CSPP1* (top) and of the fetus referred for retrognathia, complex heart defect, and small stomach with a pathogenic truncating homozygous *EFEMP2* variant associated with a truncating homozygous variant of unknown significance in *RAG1* (middle and bottom).

Between June 2019 and November 2021, we prospectively included 150 pregnancies from 19 different French centers (which included those on Réunion Island). Of the 150 couples, 111 (74%) were Caucasian and 6 (4%) were consanguineous. The term for pregnancies upon fetal sampling ranged from 10 to 31 WG (mean: 20 WG). Fetal ultrasound showed at least two major anomalies in 57/150 pregnancies (38%), one major and at least one minor anomaly in 60/150 pregnancies (40%), and one anomaly (major or minor) with a strong suspicion of genetic cause in 33/150 pregnancies (22%; Figure 1A). The ultrasound results included a wide spectrum of signs ranging from amniotic fluid anomaly in 14/150 pregnancies (9.3%) to visceral malformation in 62/150 pregnancies (41.3%) (Figure 1B). Isolated hygroma was seen on ultrasound in one consanguineous couple.

### 3.2 Molecular results

**FIGURE 2:**
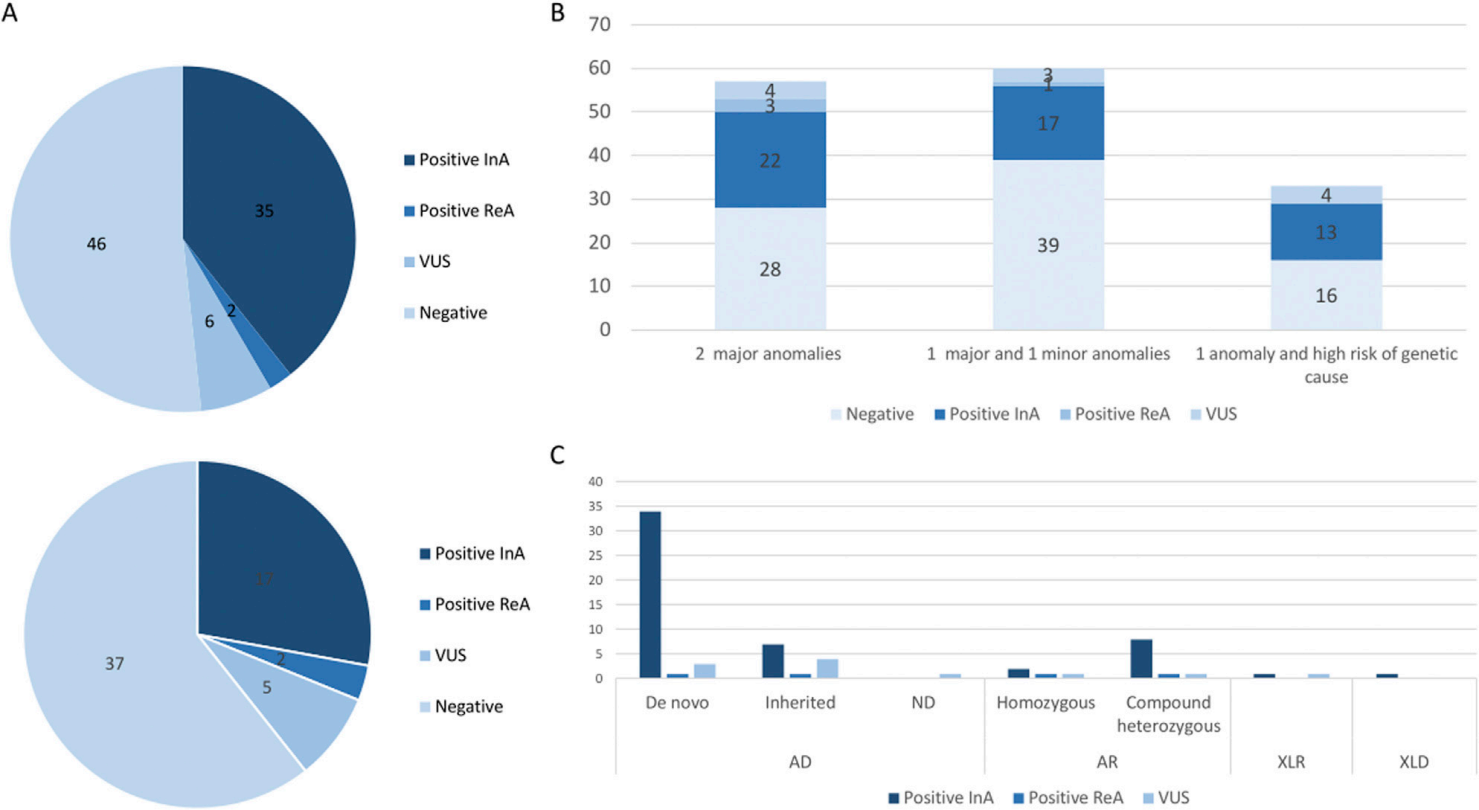
**(A)** Molecular results in the first-line trio-ES subgroup (top) and in the second-line trio-ES subgroup (below). CNV, copy number variant; InA, initial analysis; ReA, reanalysis; SNV, single nucleotide variant; VUS, variant of unknown significance. **(B)** Histograms of the molecular results stratified by a clinical subgroup, namely, two major anomalies, one major and one minor anomaly, and one anomaly with a strong suspicion of a genetic disorder. **(C)** Mode of inheritance for the identified variants and molecular results. AD, autosomal dominant; AR, autosomal recessive; FL, first-line analysis; SL, second-line analysis; VUS, variant of unknown significance; XLD, X-linked dominant; XLR, X-linked recessive.

First-line trio-ES diagnostic strategy was performed in 89/150 fetuses. A causative molecular diagnosis (likely pathogenic or pathogenic variants) was identified in 35/89 fetuses in the initial analysis (39%; Figure 2A). CNV identification was concordant between trio-ES and CMA. Second-line trio-ES was performed in 61/150 fetuses, and a causative molecular diagnosis (likely pathogenic or pathogenic variants) was identified in 17/61 fetuses in the initial analysis (27%; Figure 2A). No additional causative CNV was identified by trio-ES. The diagnostic yield was the highest in the “2 major anomalies” subgroup with 22/57 (38%) fetuses being diagnosed, rising to 25/57 (43%) with positive reanalysis included (Figure 2B).

In total, trio-ES identified a causative molecular diagnosis (likely pathogenic or pathogenic variants) in the initial analysis in 52/150 fetuses (34%), which included SNV/indel in 43/52 (83%), CNV in 8/52 (15%), and SNV/CNV in 1/52 (2%; Table 1). Pathogenic variants involved in autosomal dominant disorders were identified in 40/52 fetuses, which included *de novo* (32/40) or inherited (6/40) variants and *de novo* mosaic CNV (2/40). The six inherited SNVs were transmitted by a symptomatic parent (*COL1A2*, *IGF1R*, and *TBX3*) or an asymptomatic parent (*ACTB*, *EYA1*, and *GREB1L*). Pathogenic variants involved in recessive disorders were identified in 10/52 fetuses, which included compound heterozygous SNV/indel variants (8/10) and homozygous SNVs (2/10). Pathogenic variants involved in X-linked dominant and recessive disorders were identified in 2/52 fetuses, which included one hemizygous SNV and one heterozygous SNV (Figure 2C). Of note, the causative variants in genes implicated in RASopathies were identified in 7/52 fetuses (13%). All seven fetuses had at least four US signs (6/7 with polyhydramnios, 5/7 with macrosomia, and 4/7 with renal anomalies), leading to ToP in 5/7 (4/5 before molecular results).

**TABLE 1:** Positive molecular results and variants of unknown significance identified in the 150 fetuses of the cohort. *, positive reclassification; **, negative reclassification; ***, gene not involved in human disorder; #, fetus with a positive variant and variant of unknown significance.

Fetus	Gene	Variant segregation	Inheritance	Genomic position (hg19)	Protein
***Positive variants in ES and CMA in parallel***					
1	*ACTB*	Paternal mosaicism	Autosomal dominant	chr7:g.5568935C>A	*p*. (Gly74Cys)
2	*ANKRD11*	*De novo*	Autosomal dominant	chr16:g.89349019G>A	*p*. (Arg1311*)
3	*COL4A1*	*De novo*	Autosomal dominant	chr13:g.110827050C>A	*p*. (Gly1082Val)
4	*COL4A1*	*De novo*	Autosomal dominant	chr13:g.110804766C>T	*p*. (Glu1615Lys)
5	*DLL1*	*De novo*	Autosomal dominant	chr6:g.170598799delG	*p*. (Pro51Hisfs*72)
6	*EYA1*	Maternally inherited	Autosomal dominant	chr8:g.72211426G>A	*p*. (Gln228*)
7	*FGFR3*	*De novo*	Autosomal dominant	chr4:g.1806119G>A	*p*. (Gly380Arg)
8	*FLT4*	*De novo*	Autosomal dominant	chr5:g.180040110C>T	*p*. (Gly1111Glu)
9	*GRIN2B*	*De novo*	Autosomal dominant	chr12:g.13717487_13717488del	*p*. (His895Leufs*15)
10	*GUSB*	Maternally inherited	Autosomal recessive	chr7:g.65439612C>T	*p*. (Arg382His)
		Paternally inherited		chr7:g.65444769G>A	*p*. (Leu176Phe)
11	*HRAS*	*De novo*	Autosomal dominant	chr11:g.534,287_534288delinsAA	*p*. (Gly12Val)
12	*IGF1R*	Maternally inherited	Autosomal dominant	chr15:g.99459270_99459271dup	*p*. (Pro637Cysfs*6)
13	*L1CAM*	Maternally inherited	X-linked recessive	chrX:g.153135930G>A	*p*. (Pro240Leu)
14	*MRPS22*	Paternally inherited	Autosomal recessive	chr3:g.139067142_139067143ins	*p*. (Ile161Asnfs*4)
		Maternally inherited		chr3:g.139069025G>A	*p*. (Arg170His)
15	*NHS*	Maternally inherited	X-linked dominant	chrX:g. [17393987C>G; 17393989del]	*p*. [(Pro36Arg); (Leu38Cysfs*158)]
16	*NIPBL*	*De novo*	Autosomal dominant	chr5:g.37057351C>T	*p*. (Gln2443*)
17	*PTPN11*	*De novo*	Autosomal dominant	chr12:g.112915523A>G	*p*. (Asn308Asp)
18	*SLC26A2*	Paternally and maternally inherited	Autosomal recessive	chr5:g.149359991C>T	*p*. (Arg279Trp)
19	*SMO*	Maternally inherited	Autosomal recessive	chr7:g.128845457T>C	*p*. (Phe252Leu)
		Paternally inherited		chr7:g.128846362C>T	*p*. (Arg400Cys)
20	*TBX3*	Paternally inherited	Autosomal dominant	chr12:g.115112386_115112393dup	*p*. (Glu452Glyfs*183)
21	*TSEN54*	Maternally inherited	Autosomal recessive	chr17:g.73518081G>T	*p*. (Ala307Ser)
		Paternally inherited		chr17:g.73518112delC	*p*. (Pro318Glnfs*24)
22	*GREB1L*	Maternally inherited	Autosomal dominant	chr18:g.19053061G>	*p*. (Arg751His)
22	Del 17q12	Maternally inherited	Autosomal dominant	17q12 (34842542–36104877)x1	ND
23	*CREBBP*	*De novo*	Autosomal dominant	chr16:g.3843495G>A	*p*. (Arg370*)
24	*TUBB*	*De novo*	Autosomal dominant	chr6:g.30691800A>G	*p*. (Met321Val)
25	*PTPN11*	*De novo*	Autosomal dominant	chr12:g.112910835A>G	*p*. (Ile282Val)
26	*NFIA*	*De novo*	Autosomal dominant	chr1:g.61554154C>T	*p*. (Arg121Cys)
27	*ZNF148*	*De novo*	Autosomal dominant	chr3:g.124951946G>A	*p*. (Gln542*)
28	del16p13.3	*De novo*	Autosomal dominant	16p13.3 (3767420–3860782)x1	ND
29	del17q25.3	*De novo*	Autosomal dominant	17q25.3 (79539041–81052322)x1	ND
30	del22q11.21	*De novo*	Autosomal dominant	22q11.2 (18893886–21386103)x1	ND
31	dup15q11.2	*De novo*	Autosomal dominant	15q11.2 (22833523–25223593)x3	ND
32	Trisomy 14	*De novo*—somatic mosaicism	Autosomal dominant	ND	ND
33	Trisomy 17	*De novo*—somatic mosaicism	Autosomal dominant	ND	ND
34	Trisomy 18	*De novo*	Autosomal dominant	ND	ND
35	Tetrasomy 12p	*De novo*	Somatic mosaicism	12p11.1-p12.33 (176047–34179852)x4	ND
***Positive variants in ES after normal CMA***					
1	*ASCC1#*	Paternally and maternally inherited	Autosomal recessive	chr10:g.73970545dup	*p*. (Glu53Glyfs*19)
2	*ASXL1*	*De novo*	Autosomal dominant	chr20:g.31022449dup	*p*. (Gly646Trpfs*12)
3	*BRAF*	*De novo*	Autosomal dominant	chr7:g.140501302T>C	*p*. (Gln257Arg)
4	*COL1A2*	Maternally inherited	Autosomal dominant	chr7:g.94053703G>C	*p*. (Gly874Ala)
5	*DYNC2H1*	Paternally inherited	Autosomal recessive	chr11:g.103091449A>G	*p*. (Asp3015Gly)
		Maternally inherited		chr11:g.103124080del	*p*. (Leu3370Cysfs*35)
6	*GNB2*	*De novo*	Autosomal dominant	chr7:g.100275036A>G	*p*. (Lys89Glu)
7	*KAT6B*	*De novo*	Autosomal dominant	chr10:g.76789043_76789052del	*p*. (Ser1487Argfs*59)
8	*KMT2D*	*De novo*	Autosomal dominant	chr12:g.49445823delG	*p*. (Pro548Hisfs*382)
9	*KMT2D*	*De novo*	Autosomal dominant	chr12:g.49428657dup	*p*. (Leu3432Phefs*36)
10	*LINS1*	Maternally inherited	Autosomal recessive	chr15:g.101115226delT	*p*. (Glu200Lysfs*14)
		Paternally inherited		chr15:g.101115265_101115266del	*p*. (Lys186Serfs*17)
11	*MTOR*	*De novo*	Autosomal dominant	chr1:g.11189846A>C	*p*. (Phe1888Cys)
12	*NRAS*	*De novo*	Autosomal dominant	chr1:g.115258748C>G	*p*. (Gly12Arg)
13	*PGM1*	Paternally inherited	Autosomal recessive	NM_002633.2: c.423delA	*p*. (Ala142Glnfs*2)
		Maternally inherited		NM_002633.2:c.157_158delinsG	*p*. (Gln53Glyfs*15)
14	*PPM1D*	*De novo*	Autosomal dominant	chr17:g.58740467C>T	*p*. (Arg458*)
15	*SOS1*	*De novo*	Autosomal dominant	chr2:g.39250269C>T	*p*. (Gly434Arg)
16	*SOS1*	*De novo*	Autosomal dominant	chr2:g.39249914G>A	*p*. (Arg552Lys)
17	*SCYL2*	Paternally inherited	Autosomal recessive	chr12:g.100676845delG	*p*. (Asp33Metfs*13)
		Maternally inherited		chr12:g.100676924dup	*p*. (Glu60Glyfs*8)
***Variants of unknown significance in ES and CMA in parallel***					
1	NUP188*	Maternally inherited	Autosomal recessive	chr9:g.131760903G>A	*p*.?
		Paternally inherited		chr9:g.131745626_131745627delinsG	*p*. (Cys617Trpfs*2)
2	EFEMP2*	Paternally and maternally inherited	Autosomal recessive	chr11:g.65638118A>G	*p*. (Cys127Arg)
	*RAG1*	Paternally and maternally inherited	Autosomal recessive	chr11:g.36596621C>G	*p*. (Tyr589*)
3	*CUX1*	Maternally inherited	Autosomal dominant	chr7:g.101921327C>A	*p*. (Tyr541*)
4	*DLL1*	Maternally inherited	Autosomal dominant	chr6:g.170597444C>A	*p*. (Gly185*)
5	*DNAH11*	Maternally inherited	Autosomal recessive	chr7:g.21627820G>T	*p*.?
		Paternally inherited		chr7:g.21856224G>A	*p*. (Arg3491His)
6	*GREB1L*	Maternally inherited	Autosomal dominant	chr18:g.19019482A>T	*p*. (Asp278Val)
7	del1q21.1	ND	Autosomal dominant	1q21.1 (145414780–145826931)x1	ND
8	del6p21.32 (BRD2***)	*De novo*	Autosomal dominant	6p21.32 (32940674–32947911)x1	ND
***Variants of unknown significance in ES after normal CMA***					
1	*CSPP1#*	Paternally and maternally inherited	Autosomal recessive	chr8:g.67986545_67986546del	*p*. (Lys56Serfs*6)
2	FGF8*	Paternally inherited	Autosomal dominant	chr10:g.103530204C>T	*p*. (Arg195Gln)
3	MYCN*	*De novo*	Autosomal dominant	chr2:g.16082365C>T	*p*. (Pro60Leu)
4	dupXq28 (IDS**)	Maternally inherited	X-linked recessive	Xq28 (148564275–148798438)x3	ND
5	KAT7***	*De novo*	Autosomal dominant	chr17:g.47900657A>G	*p*.Ser494Gly
6	*KMT2E*	Maternally inherited	Autosomal dominant	chr7:g.104753422del	*p*. (His1740Profs*132)
7	*LZTR1*	*De novo*	Autosomal dominant	chr22:g.21342341A>G	*p*. (Asn148Ser)
	dup1q21.1-1q21.2	*De novo*	Autosomal dominant	1q21.1q21.2 (146397357–148344744)x3	ND

In one of the 52 fetuses, a dual diagnosis was obtained with the identification of a *GREB1L* pathogenic missense variant associated with a partial heterozygous deletion of chromosome 17 encompassing *HNF1B*.

In 16/150 fetuses (11%), trio-ES identified interesting VUS (Table 1). Variants in 3/16 fetuses were returned to the clinician during pregnancy asking for additional phenotypical features and/or to share data, leading to the reclassification as causative. For a *FGF8* sporadic missense variant, brain MRI identified lobar holoprosencephaly, supporting the pathogenicity of the variant and leading to ToP because of the poor neurodevelopmental prognosis. For a *MYCN* sporadic missense variant, only one living patient had previously been reported with a similar phenotype and a causative missense variant located in the same protein region (Kato et al., 2019). ToP was performed because of the MCA phenotype being associated with bilateral postaxial polydactyly, macrocephaly (99th centile), lateral ventricles at the normal limits, and hydramnios. Fetal autopsy allowed specifying the phenotype and international data-sharing looked for recurrence and genotype–phenotype correlation. Ultimately, this team performed functional assays that confirmed the pathogenic role of our variant after pregnancy outcome (manuscript in progress). For an *EFEMP2* homozygous missense variant, postnatal clinical examination confirmed *cutis laxa*, with the knowledge of a similar phenotype in a previously deceased fetus, highly suggestive of an autosomal recessive disorder in a consanguineous family. In 1/16 fetuses, additional investigations led to reclassify the VUS as likely benign. A 234.16 Kb maternally inherited duplication, located in Xq28, possibly resulted in an *IDS* complex mechanism leading to loss of function (Zanetti et al., 2014; Lin et al., 2019), but iduronate-sulfatase enzymatic activity was normal. In 5/16 fetuses with interesting VUS, additional investigations (reverse phenotyping of the carrying asymptomatic parent) were not contributive to variant reclassification (*CUX1*, *DLL1*, *KMT2E*, and *DNAH11*).

In addition, trio-ES identified two CNVs classified as “Variable Expression and Incomplete Penetrance” associated with an increased risk of neurodevelopmental disorders (Table 1). These were not reported to the clinicians.

In two fetuses with a causative molecular diagnosis (*EFEMP2*, *ASCC1*), trio-ES also identified noteworthy pathogenic variants not linked to the prenatal clinical presentation (Table 1). In the first fetus with the causative homozygous *EFEMP2* variant, a homozygous pathogenic truncating *RAG1* variant was also identified. Causative biallelic *RAG1* truncating variants have been implicated in a postnatal spectrum of severe immunological disorders (Meshaal et al., 2019). In the second fetus (15 WG) with isolated persistent nuchal translucency and a causative homozygous *ASCC1* variant, a homozygous pathogenic truncating *CSPP1* variant was also identified. Causative biallelic *CSPP1* variants have been implicated in Joubert syndrome (MIM:615636) (Tuz et al., 2014). Since the fetus was addressed in the early stages of pregnancy, the phenotypical symptoms of Joubert syndrome were undetectable. Despite the impossibility in considering the *RAG1* and *CSPP1* pathogenic variants as causative factors for the prenatal presentation, both results were returned to the clinicians because of the importance for genetic counseling due to the autosomal recessive mode of inheritance.

After the publication of the novel implication of *NUP188* in a human disorder, the targeted reanalysis of a fetus in our database identified causative compound heterozygous truncating variants after the outcome of pregnancy, 5 months after the first report (Muir et al., 2020).

Finally, considering the initial positive diagnosis and VUS reclassification, a causative diagnosis was made in 56/150 pregnancies (37%). The molecular results and associated phenotypes are described in Supplementary Table S1.

### 3.3 Analytical stages

**FIGURE 3:**
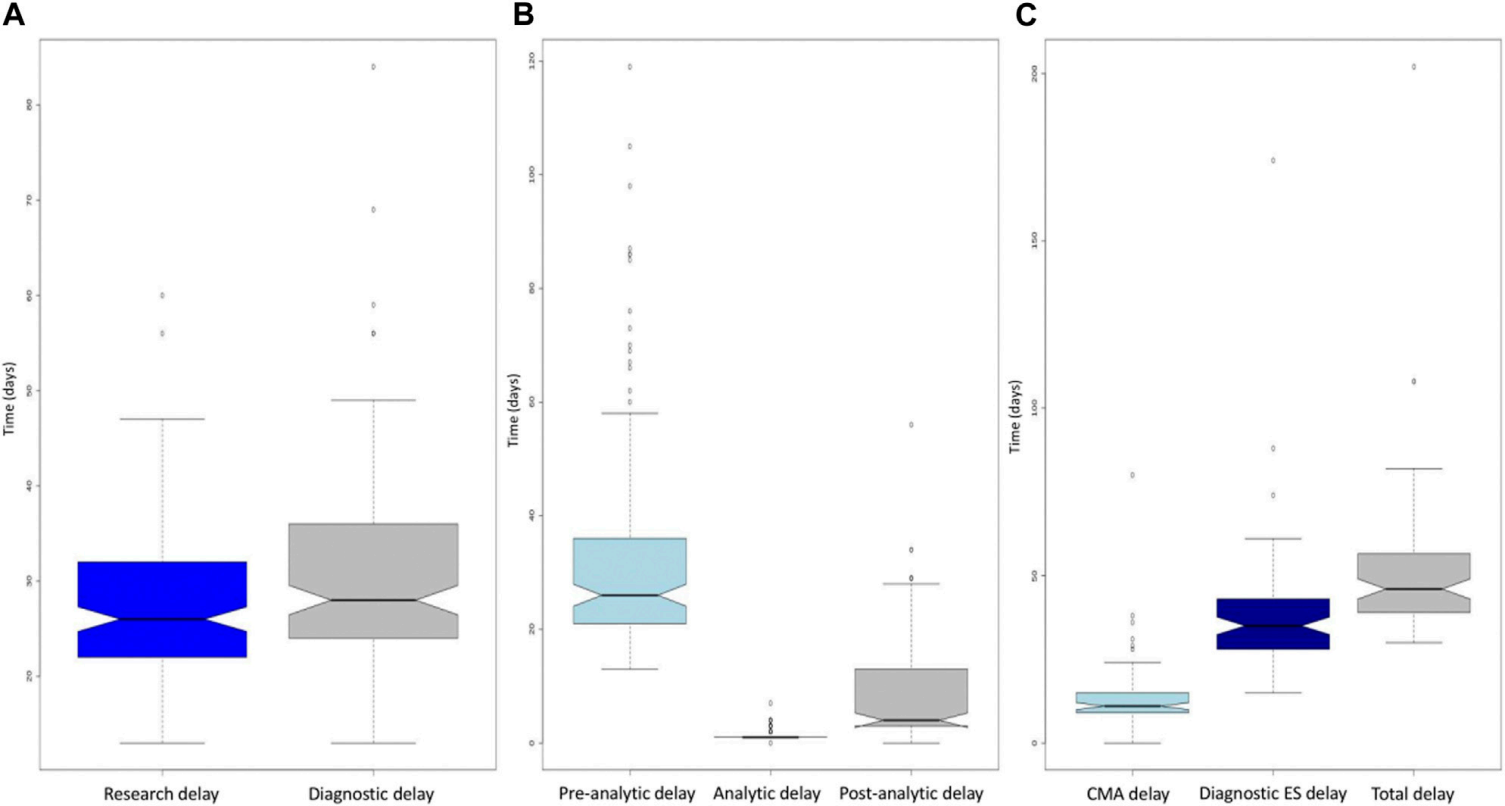
**(A)** Boxplot of the time required to obtain the report without confirmation (dark blue) and with confirmation (gray) for the entire cohort. The diagnosis delay boxplot is above the research delay one, corresponding to the mandatory diagnostic validation. **(B)** Boxplot of the pre-analytical (light blue), analytical (dark blue), and post-analytical (gray) turnaround times for the entire cohort. Note the minimal dispersion of the analytical boxplot when compared to the pre- and post-analytical ones. **(C)** Boxplots of the turnaround times for CMA (light blue), exome (dark blue), and combined CMA and exome (gray). CMA, chromosomal microarray. For the three boxplots, the threshold represents the minimum value, first quartile, mean, third quartile, and maximum. The dots represent outliers.

For the 150 fetuses, the median duration from the reception of the three samples to the emailing of the molecular report before Sanger confirmation was 26 days [13–60] (Figure 3A). The overall median duration which included Sanger confirmation was 28 days [13–84] for the 150 fetuses, 27 days [13–47] for the negative molecular results and 39 days [18–84] for the positive molecular results. This median duration was increased by 12 days because of the requirement for validation of results by a second orthogonal method to be eligible for medical ToP (Figure 3A). The median pre-analytical stage (from sampling date to raw data reception) was 26 days (13–119), the median analytical stage (from raw data reception to the available vcf file) was 1 day (1–7), and the post-analytical stage (from the available vcf file for interpretation to report) was 4 days (0–56) (Figure 3B).

For first-line trio-ES (81/150), the mean differential duration between the two results was 20 days (0–66). In two pregnancies, the trio-ES result occurred before the CMA. In all other cases, the CMA results were reported first.

### 3.4 Pregnancy outcomes

**FIGURE 4:**
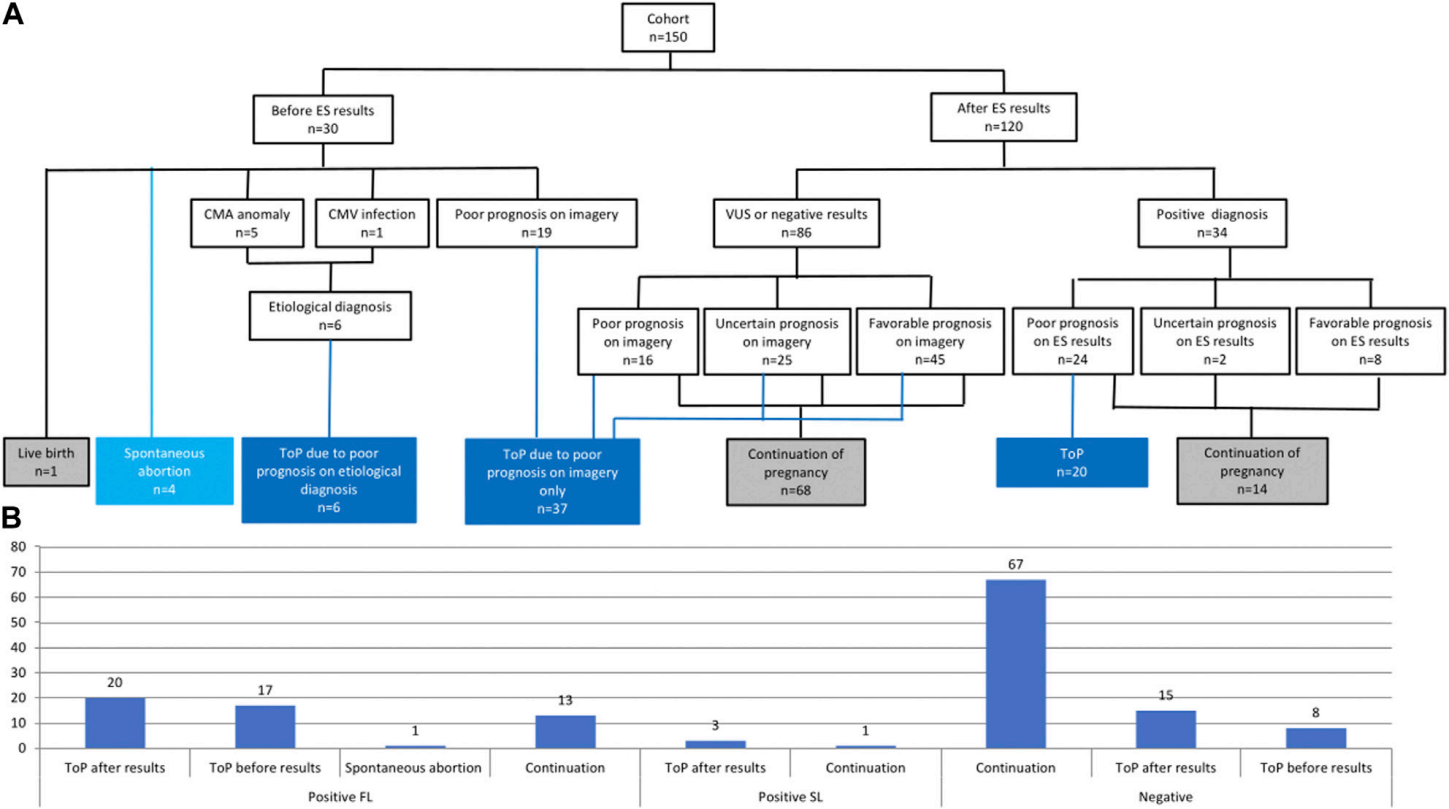
**(A)** Flowchart of pregnancy issue results separated between the availability of the ES results. CMA, chromosomal micro array; CMV, cytomegalovirus; ES, exome sequencing; ToP, termination of pregnancy; VUS, variants of unknown significance. **(B)** Pregnancy outcomes in the entire cohort depending on molecular results (positive or negative). FL, first line; SL, second line; ToP, termination of pregnancy.

Among the 150 pregnancies, 30 ended before the final molecular report was received (1 live birth, 4 spontaneous abortions, and 25 ToP) (Figure 4A). ToP was performed because the US signs progressed in favor of a poor diagnosis/prognosis (19/25), chromosomal anomalies were identified by CMA (5/25), or due to cytomegalovirus infection (1/25; Figure 4A).

Among the 120 other pregnancies in which the ES result was returned during pregnancy, two-thirds of couples (82/120) decided to continue the pregnancy, mainly because of the favorable or uncertain prognosis on imagery with a negative or VUS ES result (66/82), and also when the positive ES result gave a favorable prognosis (8/82). One-third of the couples (38/120) decided to undergo ToP procedure because of the poor prognosis associated with the ES result (20/38) or the poor or uncertain prognosis on imagery despite negative ES results (18/38) (Figure 4A). Among the 34 couples with positive ES results, 14 decided to continue the pregnancy because of a favorable prognosis on ES results (8/14), and also despite uncertain (2/14) or poor prognosis (4/4) on ES results. ES results helped considerably with parental decision-making in 94/120 pregnancies (78%). More specifically, positive ES results helped decision-making in 28/34 pregnancies (82%) since poor prognosis led to ToP in 20/34 cases and favorable prognosis led to the continuation of pregnancy in 8/34 cases. Negative or VUS ES results helped with decision-making in 66/86 pregnancies (77%), leading to continuation of pregnancy when the imagery suggested uncertain or favorable prognosis (Figure 4A).

Five of the 20 couples (25%) who obtained causative molecular diagnosis and underwent ToP asked for prenatal molecular diagnosis after confirming a subsequent pregnancy. For three families with autosomal or X-linked dominant disorders (*GREB1L*, *NHS*, and *PTPN11*), extended family segregation was performed by Sanger sequencing, resulting in a molecular diagnosis for six additional individuals.

## 4 Discussion

We report the results of the first French national multicenter study of trio-ES implementation in routine prenatal diagnosis.

This study demonstrates the feasibility of centralizing testing for multiple clinical centers in a single molecular diagnostics laboratory. A total of 150 prenatal samples were sent from 19 different French clinical centers (which included one in Reunion Island, located 9,000 km away from the laboratory) to a single laboratory that processed samples into DNA, performed bioinformatics analyses and variant interpretation, and edited the final reports. Only the sequencing step was outsourced to a single sequencing platform (from DNA to raw data). There is a financial advantage to this approach since not all laboratories can afford to invest in equipment or personnel dedicated to a fast circuit. Indeed, the discontinuous arrival of urgent samples requires either a sustained standard flow with on-demand insertion of samples or the availability of a dedicated second sequencing device used only for urgent requests, thus creating additional costs. Exclusively outsourcing data production remains economically affordable for most laboratories, which can still maintain bioinformatics and biological analysis expertise on site. A disadvantage is that additional time is then required since the outsourcing step is one of the longest in the process (median of 14 days) (Figure 3A). Although raw data outsourcing appears fast, reporting time for trio-ES could be reduced with a local solution. Sanger confirmation is the second longest step in the process (median of 14 days) (Figure 3A). This could call into question the need to confirm trio-ES in the context of pregnancy when quality metrics are met. Indeed, Sanger confirmation of variants identified by NGS seems to have equal or limited utility (Beck et al., 2016; Fridman et al., 2021). Information about Sanger validation is available in only 8 of the 11 published prenatal studies (Supplementary Table S2): 5 studies used it in all cases, 2 upon inheritance or due to quality metrics, and 1 in no case. Nevertheless, a final molecular report was returned with a median duration of 28 days, which remains compatible with decision-making during pregnancy. In one case, the total duration was of 84 days. When looking back at the data, this outlier could be explained by several accumulative factors, namely, i) the receipt of the sample in mid-December, during the holiday period, with insufficient staff, ii) the period of 2020, when France was in the second stage of the COVID-19 epidemic, and iii) national curfew imposed in mid-January 2021. All these factors forced the actors of this project to adapt to the exceptional circumstances in a degraded mode and to urgently set up new organizations, which delayed the processing of this particular sample.

The overall diagnostic yield of first-line trio-ES in our cohort (41%) reflects the rate that could be expected in routine prenatal diagnosis on malformations detected by prenatal ultrasound. While this rate is higher than in the larger published cohorts (Supplementary Table S2), studies with a diagnostic yield inferior to 20% performed on larger cohorts might be explained by less severe US anomalies, particularly isolated hygroma or increased nuchal translucency (Best et al., 2018; Ferretti et al., 2019). For these reasons, our study included only fetuses with at least two US anomalies or with one US anomaly associated with a minor or major anomaly that is known to be frequently linked to a genetic etiology, excluding isolated hygroma or increased nuchal translucency. Finally, the diagnostic yield ranged from 24% to 33% in the cohorts with similar inclusion criteria (Supplementary Table S2), which suggests that prenatal ES should not be employed in cases of isolated hygroma or increased nuchal translucency (Mellis et al., 2022; Pauta et al., 2022).

Interestingly, variants involved in RASopathies were detected in 13% of our cohort, which is consistent with previous prenatal cohorts suspected of such syndromes [diagnostic yield ranging from 9.5% to 14% (Stuurman et al., 2019; Mangels et al., 2021; Scott et al., 2021)].

*De novo* variants with an autosomal mode of inheritance accounted for the majority of causative diagnoses (32/52; 61%), which included 14 missense variants that would have been difficult to interpret without parental segregation. Therefore, a trio-based ES strategy should be favored in a context of prenatal diagnosis, even if the cost is higher than that of solo-based strategy. Indeed, the major interest of trio-based ES remains the rapid identification of sporadic variants (Gabriel et al., 2021). Moreover, trio-ES makes it possible to determine the phase of biallelic compound heterozygous variants. It also helps to highlight interesting VUS such as the *MYCN* missense variant, which was retained because of *de novo* occurrence and the absence from the Genome Aggregation Database. *MYCN* loss-of-function variants are known to be implicated in the Feingold disorder (microcephaly and absent/hypoplastic phalanx), with a mirror phenotype of the fetus (macrocephaly and polydactyly). One living individual has been reported with a similar phenotype and close missense variant (Richards et al., 2015). Data sharing and functional studies led to reclassify this variant as causative in a new *MYCN*-related phenotype (manuscript in progress).

It is also important to keep in mind that the extreme spectrum of Mendelian disorders remains elusive in prenatal settings, with variant interpretation based mostly on US signs and sometimes X-rays or MRI. Since some organs are in formation and maturation during fetal development, the identification of molecular causes and genotype–phenotype correlations can be difficult, requiring additional imaging/tests. For example, for the *FGF8* missense variant, brain MRI subsequently confirmed complete lobar holoprosencephaly, further confirming the pathogenicity of the variant. While additional tests can validate the pathogenicity of a variant, some results can also exclude them, for instance, the normal iduronate-sulfatase activity that excluded the pathogenicity of the *IDS* variant.

The diagnostic yield of first-line trio-ES was 9% for causative CNV (8/89), similar to current prenatal CMA results since pathogenic CNV were identified in 3%–6.5% of the fetuses with normal karyotypes (Callaway et al., 2013). Moreover, CMA and trio-ES were fully concordant for causal CNV identification, which may put the role of CMA in prenatal health up for debate. Indeed, if CMA had been performed as a first-line test, trio-ES would have been delayed by several days or weeks for the large majority of couples with normal CMA results (75/80; 93%). In addition, there is a risk of not performing ES after positive CMA, ruling out the possibility in identifying dual diagnosis such as the 17q12 deletion and a pathogenic inherited missense *GREB1L* in a fetus with polyhydramnios and enlarged kidneys. Since the deletion encompasses *HNF1B*, which can involve renal cysts, the *GREB1L* variant would have been missed if the CMA had been performed before trio-ES. The main advantage of CMA remains that it has faster processing time than ES (CMA reports emitted faster than ES-trio in 97% cases). In the prenatal setting, performing trio-ES as a first-line test concomitantly to CMA or CMA after negative trio-ES (depending on the CNV detection pipeline used for ES) could be suggested.

Despite concerns about the time required for the analyses, almost all reports were returned before the predicted term of pregnancy (except for *NUP188*, which was identified after ToP), which means that they could theoretically have been used in prenatal management. Nevertheless, 23/150 pregnancies (15%) were terminated before the molecular report was obtained because an unfavorable fetal prognosis was identified on US, emphasizing the need to shorten turnaround times. However, it is worth reassessing the initial indications for prenatal ES. Indeed, the analysis of ES data with detailed phenotyping after fetal autopsy and without the pressure of an emergency context would be easier for clinical laboratories than performing prenatal ES. The indications of prenatal ES should be therefore discussed by a multidisciplinary team and offered to couples when fetal prognosis based on ultrasound features remains uncertain and a molecular diagnosis would genuinely help with decision-making. For example, in a 25 WG fetus with a highly variable fetal prognosis (corpus callosum agenesis and ventriculomegaly without any other malformations) (Yeh et al., 2018; Bernardes da Cunha et al., 2021), ES evidenced a causative maternally inherited hemizygous *L1CAM* missense variant with a poor neurodevelopmental prognosis (MIM:304100), leading to ToP after the molecular result. The heterozygous mother may also benefit from early prenatal diagnosis for future pregnancies. In a 30 WG fetus with intrauterine growth restriction and hypotelorism leading to unknown fetal prognosis, ES evidenced a causative heterozygous *IGF1R* truncating variant inherited from the mother, who was of short stature. This finding confirmed the diagnosis of resistance to insulin-like growth factor I (MIM:270450), which has a favorable neurodevelopmental prognosis, and the pregnancy was therefore maintained (Supplementary Table S1).

Altogether, diagnostic results were returned to 120/150 (80%) couples with ongoing pregnancies, which included 34 with a causative diagnosis and 86 with negative or VUS results. In 28/34 fetuses with causative diagnosis (82%), the results were helpful for pregnancy management: a poor prognosis led to ToP while a reassuring prognosis led to the continuation of pregnancy with monitoring. Among the 86 fetuses with no causal diagnosis, parents tended to continue with the pregnancy (68/86; 79%). Altogether, the results had a potential effect on pregnancy management in 78% of cases, a rate which is similar to a previously published report (67%) (Dempsey et al., 2021).

The VUS of interest were returned to couples in 17/150 pregnancies (11%) because their implication in the phenotype seemed very likely and/or additional investigations could be performed to confirm or rule out pathogenicity. Finally, 4/17 VUS were reclassified as pathogenic and 1/17 as likely benign. Returning VUS to the couples in prenatal setting remains difficult due to the uncertain involvement of these variants in fetal phenotypes (Narayanan et al., 2018; Richardson and Ormond, 2018; Werner-Lin et al., 2019; Harding et al., 2020). Therefore, it will be important to establish guidelines to specify the VUS that should be returned to couples. VUS should also be discussed on a case-by-case basis when laboratories do not have clear policies about reporting during pregnancy. This question appears to be up for debate since two previous studies systematically reported VUS, whereas three did not, and five studies (including ours) decided on a case-by-case basis, highlighting the complexity of managing these data in the prenatal period and the need for consensual guidelines (Supplementary Table S2).

Moreover, two cases involving consanguineous couples highlighted particular difficulties when diagnosing several autosomal recessive syndromes with unusual or undetectable signs on prenatal US (*EFEMP2*-*RAG1* and *ASCC1*-*CSPP1*). Indeed, *RAG1* is involved in a severe spectrum of immunodeficiencies that can only be detected after birth (MIM:601457), while *CSPP1* is involved in Joubert syndrome (MIM:615636) for which prenatal signs cannot be detected by US at early stages. These examples show the potential interest of detecting variants that do not account for prenatal US signs but could be considered actionable incidental findings for genetic counseling (25% chance of recurrence for subsequent pregnancies). This also emphasizes the need for laboratories performing prenatal ES to carefully establish their policies regarding incidental findings (Vora et al., 2020; Basel-Salmon and Sukenik-Halevy, 2022; Vears and Amor, 2022).

In conclusion, prenatal trio-ES provides a considerable diagnostic yield in fetuses with US abnormalities and appears significantly helpful for couples seeking guidance regarding pregnancy management. It could be therefore routinely implemented when the fetal prognosis remains uncertain on US features and molecular diagnosis would support decision-making. However, the indications should be discussed by a multidisciplinary team. The complete concordance between trio-ES and CMA for diagnosis of CNV suggests that trio-ES is an appropriate first-line test to obtain a causal diagnosis as quickly as possible. Medico-economic studies would now be useful to better understand the cost–benefit ratio of such a rapid prenatal trio strategy in fetuses with US signs.

## Data Availability

The original contributions presented in the study are publicly available. This data can be found here:https://www.ncbi.nlm.nih.gov/clinvar/. Accession number: SUB12873630.

## Supplementary material

The Supplementary Material for this article can be found online at: https://www.frontiersin.org/articles/10.3389/fgene.2023.1099995/full#supplementary-material

Supplementary Table S1 Entire fetal cohort with clinical signs, CMA subgroup, molecular results and pregnancy management. CC: Corpus Callosum; CCA: Corpus Callosum agenesis; CMA: Chromosomal microarray; IUGR: Intrauterine growth restriction; INT: Increased nuchal translucency; MRI: Magnetic resonance imaging; PH: Polyhydramnios; US: Ultrasound.

Supplementary Table S2 Previous studies with fetal cohort, genetic investigations, molecular results, variants of unknown significance and Sanger confirmation policy, and turnaround time. CC: Corpus Callosum; CMA: Chromosomal microarray; ES: Exome sequencing; NR: Not reported; QC: quality control; VUS: Variants of unknown significance.
